# Efficacy of Sotrovimab (SOT), Molnupiravir (MOL), and Nirmatrelvir/Ritponavir (N/R) and Tolerability of Molnupiravir in Outpatients at High Risk for Severe COVID-19

**DOI:** 10.3390/v15051181

**Published:** 2023-05-17

**Authors:** Victoria Kauer, David Totschnig, Ferdinand Waldenberger, Max Augustin, Mario Karolyi, Michelle Nägeli, Christoph Wenisch, Alexander Zoufaly

**Affiliations:** 1 Vienna Healthcare Group, Department of Medicine IV, Klinik Favoriten, Kundratstraße 3, 1100 Vienna, Austria; david.totschnig@gmail.com (D.T.); max.augustin@gmx.net (M.A.); mario.karolyi@gmx.at (M.K.); mnaegeli@bidmc.harvard.edu (M.N.); christoph.wenisch@gesundheitsverbund.at (C.W.); alexander.zoufaly@gesundheitsverbund.at (A.Z.); 2 ASBÖ Arbeiter-Samariter-Bund Floridsdorf-Donaustadt, 1150 Vienna, Austria; ferdinand.waldenberger@samariter.at; 3 Department I of Internal Medicine, Medical Faculty and University Hospital Cologne, University of Cologne, 50937 Cologne, Germany; 4 Faculty of Medicine, Sigmund Freud University, 1200 Vienna, Austria

**Keywords:** COVID-19, SARS-CoV-2, coronavirus, antiviral therapy, outpatient treatment, sotrovimab, nirmatrelvir/ritonavir, molnupiravir

## Abstract

Objective: The main goal of this study was to assess the potential clinical impact of an outpatient administration of available antivirals including SOT, N/R, and MOL to COVID-19 patients at high risk for disease progression. Methods: We conducted a retrospective analysis on 2606 outpatient individuals with mild to moderate COVID-19 at risk for disease progression, hospitalization, or death. After receiving either SOT (420/2606), MOL (1788/2606), or N/R (398/2606), patients were followed-up with regarding primary (hospitalization rate) and secondary (treatment and side effects) outcomes by phone. Result: A total of 2606 patients were treated at the outpatient clinic (SOT: 420; N/R: 398; MOL: 1788). 3.2% of the SOT patients (1 ICU admission), 0.8% of the MOL patients (2 ICU admissions), and none of the N/R patients were hospitalized. 14.3% of the N/R patients reported strong to severe side effects, exceeding SOT (2.6%) and MOL (5%) patients. A reduction in COVID symptoms after the treatment was experienced by 43% of patients in both the SOT and MOL groups and by 67% of patients in the N/R group, respectively. Women had a higher chance of symptom improvement with MOL (OR 1.2, 95%CI 1.0–1.5). Conclusion: All antiviral treatment options effectively prevented hospitalization in high-risk COVID-19 patients and were well tolerated. Side effects were pronounced in patients with N/R.

## 1. Introduction

The coronavirus disease 2019 (COVID-19) pandemic rapidly spread across the globe, even among communities with a high level of preexisting immunity due to vaccination [1,2,3,4]. Efficient and safe antivirals are vital to treat severe acute respiratory syndrome coronavirus type 2 (SARS-CoV-2)-related infections as early as possible in an outpatient setting to prevent disease progression and thus hospitalization and death [5]. Sotrovimab (SOT), molnupiravir (MOL), and nirmatrelvir/ritonavir (N/R) have been shown to reduce the risk of hospitalization and COVID-19-related death. Starting all of these drugs is recommended as early as possible after infection, within the first five days after the onset of symptoms at the latest [6,7].

N/R is an approved SARS-CoV-2 (Mpro) protease inhibitor containing the active component nirmatrelvir, as well as ritonavir, a pharmaceutical enhancer [8]. It was granted Emergency Use Authorization (EUA) in December 2021 as therapy for non-hospitalized patients (adults and children of twelve years and older) [9]. SOT is a pan-sarbecovirus monoclonal antibody, administered in the form of a one-time intravenous infusion, directed against the SARS-CoV-2 virus by binding the virus’s spike protein. SOT is currently the only monocular antibody therapy approved for the Omicron COVID-19 variant [10]. N/R and SOT have been approved by the EMA for patients with COVID-19 with mild to moderate symptoms and without the need for oxygen supplementation who are at high risk for progression to severe disease. MOL acts as an orally active RdRp (RNA-dependent RNA polymerase) inhibitor to reduce viral load and has not yet been approved for use in the European Union or the United States. All of these antivirals can easily be used in outpatients with a recent SARS-CoV2 infection and can potentially reduce the rate of progression to severe COVID-19, which thereby may alleviate the pressure on overburdened hospital capacities.

Here, a retrospective single-center analysis was performed to provide real-world evidence of the antiviral efficacy and safety of sotrovimab (SOT), molnupiravir (MOL), and nirmatrelvir/ritonavir (N/R), which had gradually become clinically available and were used to prevent complications in outpatients infected with the Delta and Omicron SARS-CoV2 variants during a pandemic surge in Vienna.

## 2. Methods

### 2.1. Study Design and Outcomes

This retrospective analysis was based on data obtained by the Vienna health authorities, who receive electronic laboratory reports on all patients who test positive for SARS-CoV2 in Vienna. The study included data from patients who were recently diagnosed with SARS-CoV2 infection and deemed at high risk for severe COVID-19, who were thus invited for outpatient antiviral treatment between 2 January 2022 and 29 June 2022. The prevailing variants of the SARS-CoV-2 virus were Delta (B.1.617.2) between December 2021 and January 2022, Omicron (BA.1.) from mid-January to March 2022, Omicron (BA.2) from March to May 2022, and Omicron (BA.4/5) from May 2022 onwards. The primary study outcome was hospitalization due to COVID-19. The secondary study outcomes were the subjective effect of the different drugs, as well as treatment-associated side effects.

### 2.2. Study Population

**Figure 1:**
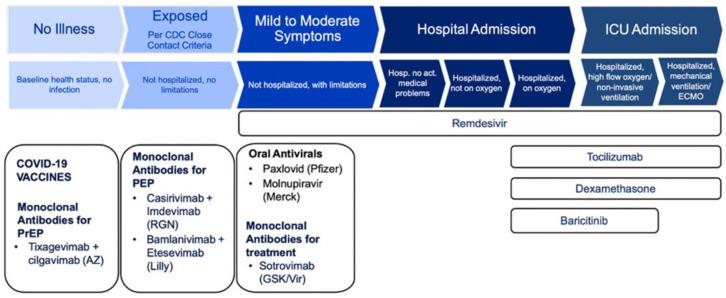
Strategy for COVID-19 prevention measures and medication.

Following a positive SARS-CoV-2 swab and electronic reporting by the laboratories, patients were contacted by telephone by the health authorities of Vienna, and those who were at high risk for disease progression, hospitalization, or death were offered outpatient oral (N/R or MOL) or intravenous (SOT) antiviral treatment, respectively. Figure 1 shows the strategy for COVI-19 prevention measures and medication used in Vienna, Austria during the time of the omicron variant.

The study population comprised individuals who had (1) confirmed SARS-CoV-2 infection, (2) received a diagnosis of COVID-19 as outpatients, (3) were assessed as being at high risk for progression to severe disease, (4) suffered from mild-to moderate symptoms, and (5) were deemed eligible to receive an antiviral therapy. High risk patients were identified based on a risk model that was developed in accordance with the national immunization board to evaluate the risk of severe COVID-19 in patients infected with SARS-CoV-2. Prioritization of the risk groups was based on the presence of one of four key elements: (1) older age, (2) no receipt of previous SARS-CoV2 vaccination, (3) immunosuppression, and (4) clinical risk factors. The most common risk factors were (1) age > 50 years, (2) obesity (BMI > 30), (3) cardiovascular diseases, (4) diabetes mellitus type 2 (DM II), (5) chronic lung and respiratory diseases, (6) chronic kidney diseases with impairment of the kidney function, (7) chronic liver disease with impaired liver function, (8) chronic psychiatric disorders, and/or (9) chronic neurological diseases.

Drug allocation was performed according to eligibility and availability criteria, as well as the patient’s preference, through telemedicine by a physician. Oral medication (MOL and N/R) was delivered to the patients’ homes. Intravenous SOT was administered at a dedicated infusion ward at the hospital (Klinik Favoriten). Eligibility criteria for the individual drugs were also dependent on supply and demand changes. For this outpatient program, SOT was available from 3 January 2022, MOL was available from 28 January 2022, and N/R was only available from the 15 March 2022 onwards until the end of the analyzed time span. In order of preference, the following therapeutics were recommended based on availability: 1. N/R, 2. SOT, 3. MOL. For each patient, a questionnaire-based follow-up (FU) interview was conducted after 28 days via telemedicine. This FU had the intention to assess the effect of the therapy forms, as well as the prevalence of adverse reactions and serious adverse events. In a set of 12 questions, patients were asked about (1) symptom start, (2) date of first positive and (3) first new negative SARS-CoV-2 PCR test, (4) hospital administration and/or (5) COVID-19-related admission to the intensive care unit (ICU), (6) side effects they noticed during treatment, and (7) their subjective well-being after drug administration. In addition, other questions mentioned included (8) adherence to medication, (9) allergic reactions to the medication, and (10) drug interactions with other medication. Concerning the subjective therapeutic effect, patients were asked (11) whether the treatment showed a good, moderate, or no effect by subjectively rating the alleviation or worsening of symptoms during treatment. Finally, patients were asked (12) to rate the side effects from a scale of 1–4 (1: severe side effects, 2: moderate, 3: mild, 4: no side effects).

### 2.3. Statistical Analysis

Data were analyzed using GraphPad Prism 9 (Dotmatics, Boston, MA, USA) and SPSS (IBM 23, USA). *p*-values of 0.05 and lower were considered as scientifically significant. Statistical parameters (value of n, statistical calculation, etc.) are stated in the figure legend. All values are represented as the median with interquartile range (IQR), unless otherwise stated. The results from the outcomes are represented as % (n/N), with N representing the patients with a complete follow-up for the corresponding question, not the patient number in total. In the MOL cohort, binomial regression models for subjective symptom improvement according to presence or absence of risk factors such as older age, pre-existing conditions, and sex were performed. Here, unadjusted risk ratios with 95% confidence intervals (CI) as well as Chi-square tests were calculated. STATA version 17.0 and GraphPad Prism (GraphPad Software, La Jolla, CA, USA) were used to compile the analyses and graph the data.

### 2.4. Ethical Considerations

The study was approved by the Institutional Review Board of the City of Vienna (EK 22-195-1022).

## 3. Results

Data from a total of 2606 patients were included in the analysis. The median age was 62 years (interquartile range (IQR) 18–101); 42% (1096/2606) of patients were 65 years of age or older and 47% (1219/2606) were women. Age ranged from 18 to 101 years old. Among the 2606 patients in the study cohort, 15% (398/2606) received at least one dose of N/R, 16% (420/2606) received SOT, and 68% (1788/2606) were treated with MOL during the study period. Considering the different treatment types, age, gender, and risk factors did not show significant variations. Demographics and patient characteristics are demonstrated in Table 1. Risk factors for severe illness were only collected in patients who received MOL. The most common risk factors in this patient collective included age over 50 years (58%), followed by cardiovascular diseases (17%), body mass index (BMI) > 30 (10%), diabetes mellitus type 2 (DM II) (7%), and chronic renal disease (2.7%).

Hospitalization rates were low in all three treatment types. In the SOT group, 3.2% (10/420) patients were later admitted to the hospital, while 0.3% (1/309) were admitted to the ICU. In comparison, 0.8% (11/1406) of the patients who received MOL were admitted to the hospital and 0.1% (1/1406) were admitted to the ICU. No patient who received N/R was admitted to hospital (0/333). In the MOL group, four patients were hospitalized due to COVID-related respiratory failure, two patients due to hypertension, four patients due to gastrointestinal symptoms, and one patient due to fatigue symptoms. In the SOT group, most patients were hospitalized due to hypotension and fatigue symptoms. Additionally, two patients were hospitalized due to a hypotensive reaction directly after SOT administration.

Of the patients who received SOT, 43% (132/309) experienced a good treatment effect after treatment, 30% (93/309) experienced a moderate treatment effect, and 27% (84/309) experienced no effect. Of the patients who received MOL, 43% (572/1334) experienced a good treatment effect during treatment, 29% (388/1334) experienced a moderate treatment effect, and 28% (374/1334) experienced no treatment effect. Of the patients who received N/R, 67% (115/227) reported a good treatment effect, 23% (39/227) experienced a moderate treatment effect, and 10% (17/1334) reported no subjective treatment effect.

Regarding the group of patients that received SOT, most patients (85% (263/309)) did not report any new symptoms after drug administration, while 15% (46/ 309) did develop new symptoms. One of these 309 patients self-reported severe symptoms (fainting) and had to be admitted to the hospital. New symptoms mostly included nausea, circulatory collapse, hypotension, vertigo, headaches, shivers, and rare cases of exanthema. In one patient, atrial fibrillation occurred.

Regarding the N/R patient collective, 47% (150) of the patients self-reported new symptoms after treatment, including the 4% (12/322) of patients who reported having experienced severe new symptoms, while 53.4% (172/322) of the patients did not show any new symptoms at all. Often mentioned symptoms included mostly gastrointestinal symptoms, foremost diarrhea, dysgeusia (metallic, bitter taste), hypertension, exanthema, circulatory problems, and headaches.

Seventy-eight percent (1048/1349) of the patients in the MOL patient collective developed no new symptoms after treatment administration. In comparison, 22% (301/1349) of the patients reported new symptoms, including 7% (23/1349) with severe new symptoms. Symptoms comprised gastrointestinal complaints (abdominal cramps, diarrhea, vomiting), blood hypertension, headaches, exanthema, itching, circulatory complaints, and vertigo. Notably, 300/1788 patients submitted a detailed description of experienced side effects; 56/300 did not experience any side effects. Proportional allocation of the new symptoms of 244/300 patients after MOL administration is presented in Table 2. Treatment of side effects was not part of the perspective of this study and such data was therefore not collected.

## 4. Secondary Outcome: Risk Stratification for MOL

Detailed risk factors for severe disease were only assessed for patients who received MOL. A risk stratification for this patient collective showed that women had a 1.2% (OR 1.0–1.5, *p* = 0.04) higher chance of a subjective treatment effect than men. Patients over the age of 60 and patients with chronic kidney disease were less likely to report symptom improvement after starting MOL, although this did not reach statistical significance (Table 3) Risk factors were not assessed for SOT and N/R, as data was not adequately collected for these two patient groups and risk stratification could not be applied.

## 5. Discussion

In this retrospective single-center analysis of high-risk COVID-19 patients with mild to moderate symptoms not requiring hospitalization, outpatient therapy with single-dose sotrovimab (SOT) or 5 days of molnupiravir (MOL) or nirmatrelvir/ritonavir (N/R) resulted in an overall low risk of disease progression and hospitalization.

Patients who received SOT had the highest hospitalization rate with 3.2% (ICU administration of 0.3%), compared to 0.8% in the MOL collective (0.1% ICU admission). None of the patients who received N/R were admitted to hospital. Based on this limited data on the safety and efficacy of oral antiviral drugs, as well as SOT in patients with COVID-19, current guidelines and the medical community of Austria are now prioritizing their distribution to those who do not require supplemental oxygen but who are at the highest risk of disease progression. The analyzed study cohort reflected such a prescription pattern in the real world, and the use of antiviral COVID-19 medication was clearly associated with a low rate of disease progression, reflected by low hospitalization rates for all three treatment options, especially for N/R.

These findings are in line with other studies. The MOVe-OUT trial by Bernal et al. included 1433 participants and indicated a lower risk of hospitalization for any cause of death until day 29 in patients receiving MOL (28 of 385 participants (7.3%) versus placebo (53 of 377 (4.1%)) [11]. Similarly, SOT in non-hospitalized patients with mild to moderate COVID-19 caused by the Delta variant reduced all-cause hospitalization lasting longer than 24 h or resulting in death (1% vs. placebo 6%) [12]. Additionally, the COMET-ICE trial, which recruited patients until April 2021, showed a relative risk reduction of 85% comparing patients who received SOT vs. a placebo group [13]. Another study by Wen et al. showed that treatment with an antiviral drug (N/V, MOL) reduced hospitalization rates by approximately 80% when compared to a placebo [3]. In comparison, the OpenSAFELY trial, a study conducted during Omicron BA.1 by Zheng et al., showed a lower risk of severe outcomes in patients at high risk of disease progression who received SOT compared to patients who received MOL [14]. The PANORAMIC trial, a multicentre trial conducted between December 2021 and April 2022 lead to the conclusion that MOL did not reduce the frequency of COVID-19-associated hospitalizations among high-risk patients [15]. This stays in contrast to a study by Arbel et al. stating that MOL did show a reduction in hospitalization in a cohort of non-hospitalized, Omicron-infected high-risk patients. However, this effect could only be noticed in patients over the age of 65 years. No effect was seen in younger patients [16]. The EPIC-HR trial resulted in an 89% reduction in risk of progression to severe COVID-19 in symptomatic patients who received N/R [9]. Additionally, Arbel et al. concluded in a study among patients of 65 years of age or older that the rate of hospitalization and death due to COVID-19 was significantly lower in patients who received N/R compared to patients who received a placebo [17].

The most common adverse events of the three oral antiviral drugs include nausea, abdominal pain and diarrhea, headache, vertigo, and circulatory complaints. In addition to these common side effects, patients who received N/R commonly reported a metallic taste that lasted for hours, which previously has been shown to occur in around 5.6% patients [8]. THE EPIC-HR trial by Hammond et al. came to the conclusion that dysgeusia and diarrhea occurred more often with N/R than with a placebo. The same study also indicates that N/R neither improves nor aggravates the occurrence of adverse events, generally stating that this oral antiviral drug is safe [9].

In comparison to N/R (14.3%), the patients who received SOT reported significantly fewer new symptoms after drug administration (2.4%). However, some of the reported symptoms were self-classified to be of greater severity, such as circulatory collapse, hypotension, and nausea, which led to hospital admission in two cases. These results are in line with the placebo-controlled, randomized COMET-ICE study, in which the safety of SOT was evaluated in 1049 non-hospitalized COVID-19 patients, recording similar rates of adverse events in the placebo (19%) group and in the group having received SOT (17%). Serious adverse events, such as hypersensitivity, infusion-related reactions, and anaphylaxis, occurred in 2% of the patient collective (compared to 6% in the placebo group), but were considered not to be related to drug intake in all cases [14]. These results are in line with our results, with most reported adverse events being linked to COVID-19 rather than to the antiviral drugs.

Patients who took MOL reported strong to severe side effects in 5.1% of cases, which is in line with other studies; a study by Painter et al., for example, stated that the occurrence of adverse events was low, and no significant difference could be found between the placebo group and the patient cohort. In this study, mild to moderate adverse events were reported in 37.5% of cases, showing results comparable to those of our analyses, with similar rates of mild to moderate adverse events (42.9%) [18]. The most common side effects represented in the study were diarrhea, nausea, headache, and abdominal pain.

Experienced subjective treatment effects of the different treatments were difficult to quantify. Nonetheless, most patients who took N/R reported a beneficial treatment and alleviation of symptoms during therapy. This can be compared to a patient collective having received MOL with a good subjective treatment effect in 42% of the cases, which is similar to patients having been treated with SOT, who experienced an alleviation in symptoms in 43% of the cases. Similar results are described in the EPIC-HR trial that showed that the efficacy of N/R was maintained in a population of participants at high risk for severe COVID-19, with N/R resulting in a risk of progression to severe COVID-19 that was 89% lower than the risk with placebo [9]. These results can be compared to the MOVe-OUT trial, in which MOL was associated with a decrease in symptom progression and the relative risk of hospitalization by 30% [11]. The superiority of N/R in comparison to MOL was underpinned by a recent study by Burdet et al., in which the risk of all-cause mortality was reduced by 24% with MOL and by 66% with N/R [19]. Nonetheless, a study comparing eight different studies suggests, citing moderate-certainty evidence from these trials, that MOL reduces the risk of hospital admission (43 fewer admissions per 1000 patients at highest risk) and the time to symptom resolution (in an average of 3.4 fewer days), while low-certainty evidence suggests a small effect on mortality (6 fewer deaths per 1000 patients). Another retrospective study concluded that initiating N/R within the first 5 days of SARS-CoV-2 infection was associated with a significantly reduced risk of progression to severe COVID-19 or mortality [20].

Limitations to this study include the lack of a control group that did not receive any antivirals. Therefore, it can only be stated that hospitalization rates were overall low after the use of antiviral drugs, but efficacy could not be assessed versus no treatment. This is of particular interest, as the Omicron VoC generally causes a milder clinical course as compared to former variants, including Alpha and Delta, which were predominant in most randomized trials concerning antiviral treatment. In addition, clinical utility of monoclonal antibodies, including SOT, is severely limited, with the emergence of some Omicron subvariants showing a high degree of immune escape [2]. At the time of the study, BA.1 was the predominant strain in Vienna, for which SOT shows neutralization capability. A further limitation of our study is the heterogeneity of completed follow-ups in the different patient cohorts as well as the different questions. No complete statements of the risk factors in the N/R and the SOT group were available; therefore, this data was not included in the study. Days until seronegativity as well as days until a negative PCR test were not reported, and this data could not be included in the study.

Future placebo-controlled, large-sampled studies will be needed to assess the short- and long-term safety and efficacy of N/R, MOL, and SOT.

## 6. Conclusions

Based on this retrospective single-center analysis providing real-world data, Sotrovimab (SOT), Molnupiravir (MOL), and Nirmatrelvir/ Ritonavir (N/R) are safe and efficacious options for treating SARS-CoV-2 infected adults at risk for disease progression, hospitalization, or death in an outpatient setting. N/R was associated with the lowest hospitalization rate but was associated with over twice as many reported adverse events as compared to the other two treatments. MOL showed a low rate of adverse events, as well as a small percentage of hospitalized cases. SOT had the lowest rate of side effects but has to be delivered intravenously, and efficacy against certain Omicron subvariants varies. Efficacy of the presented drugs might vary depending on new upcoming variants. These data are reassuring and suggest that the beneficial outcomes in terms of prevention of hospital admissions which were observed in clinical trials can be reproduced in real life situations. However, more data on other outcomes including time to complete and sustained symptom resolution, prevention of long-COVID, and faster return to work, to name a few, have yet to be convincingly proven for antiviral therapies against SARS-CoV2 infection.

## Figures and Tables

**Table 1:** Demographics and patient characteristics. MOL, Molnupiravir; N/R, Nirmatrelvir/Ritonavir; SOT, Sotrovimab; no., number; y, years; BMI, body mass index; n.p., not presentable.

Characteristic	MOL (*n* = 1788)	N/R (*n* = 398)	SOT (*n* = 420)	Total (*n* = 2606)
Female Sex—no. (%)	949 (53)	219 (55)	219 (52)	1387 (53)
Age group—no. (%)				
18–49 years	18 (4%)	48 (12%)	18 (4%)	171 (7%)
>50 years	402 (96%)	350 (88%)	402 (96%)	2435 (93%)
Median age (IQR)-y	61 (18–101)	62 (18–97)	64 (21–101)	62 (18–101)
Risk factors for severe illness—no. (%)				
BMI > 30	167 (10)	n.p.	n.p.	n.p.
Cerebrovascular disease	23 (1)	n.p.	n.p.	n.p.
Cardiovascular disease	280 (17)	n.p.	n.p.	n.p.
DM II	117 (7)	n.p	n.p.	n.p.
Chronic renal disease	47 (3)	n.p.	n.p.	n.p.
Chronic liver disease	24 (1)	n.p.	n.p.	n.p.
Immunocompromised patients	28 (1)	n.p.	n.p.	n.p.
Malignancies	36 (1)	n.p.	n.p..	n.p.

**Table 2:** New symptoms after MOL administration.

Diarrhea	59 (24%)
Nausea	52 (21%)
Headache	40 (16%)
Abdominal pain	37 (15%)
Exanthema	33 (14%)
Dizziness	31 (13%)
Fatigue	12 (5%)
Pruritus	8 (3%)
Elevated Blood Pressure	8 (3%)
Coughing	5 (2%)
Shortness of Breath	3 (1%)
Obstipation	2 (1%)
Fever	1 (0.5%)

**Table 3:** Risk factors for subjective symptom improvement.

	Risk Factors for Symptom Improvement	
	RR (95% CI)	*p*-value
female sex	1.2 (1.0–1.5)	0.04
age > 50	0.98 (0.82–1.2)	0.82
age > 60	0.8 (0.67–0.96)	0.08
BMI > 30	1.2 (0.91–1.6)	0.19
cerebrovascular disease	1.3 (0.6–2.2)	0.54
chronic liver disease	1.3 (0.6–2.2)	0.72
chronic kidney disease	0.41 (0.16–0.96)	0.05
DM II	1.0 (0.71–1.5)	0.86
immunosuppressive therapy	1.2 (0.6–2.1)	0.59
cardiovascular disease	1.0 (0.79–1.3)	0.92
cancer	1.1 (0.56–1.8)	0.85

## Data Availability

Data is available at Klinik Favoriten. Please contact the corresponding author.
